# Association of NETs Markers with Clinical and Radiological Outcomes in Patients with Acute Ischemic Stroke Undergoing Thrombectomy: Does Heparin Treatment Modify This?

**DOI:** 10.1007/s12975-025-01362-0

**Published:** 2025-06-26

**Authors:** Aarazo Barakzie, Wouter van der Steen, A. J. Gerard Jansen, Bob Roozenbeek, Samantha J. Donkel, Aad van der Lugt, Hester Lingsma, Diederik W. J. Dippel, Hugo ten Cate, Moniek P. M. de Maat

**Affiliations:** 1 Department of Hematology, Erasmus MC Cardiovascular Institute, University Medical Center Rotterdam, Dr. Molewaterplein 40, 3015 GD Rotterdam, The Netherlands; 2 Department of Neurology, Erasmus MC, University Medical Center Rotterdam, Rotterdam, The Netherlands; 3 Department of Public Health, Erasmus MC, University Medical Center Rotterdam, Rotterdam, The Netherlands; 4 Department of Radiology and Nuclear Medicine, Erasmus MC, University Medical Center Rotterdam, Rotterdam, The Netherlands; 5 Department of Biochemistry, CARIM School for Cardiovascular Diseases, Maastricht University Medical Centre, Maastricht, The Netherlands; 6 Thrombosis Expertise Center and Department of Internal Medicine, Maastricht University Medical Centre, Maastricht, The Netherlands

**Keywords:** Ischemic stroke, NETs markers, Inflammation, Coagulation, Unfractionated heparin, Endovascular thrombectomy, Stroke severity, Functional outcome, Infarct size

## Abstract

The aim of this study is to investigate the association of neutrophil extracellular traps (NETs) markers with clinical and radiological outcomes in acute ischemic stroke (AIS) patients undergoing endovascular thrombectomy (EVT) and assess the effect of periprocedural heparin during EVT on NETs markers and their association with outcomes. From 198 AIS patients included in the MRCLEAN-MED trial, randomized to receive EVT with (*N* = 104) or without (*N* = 94) low-dose unfractionated heparin (5000 IU bolus followed by 500 IU/h for 6 h, *n* = 104), blood samples were collected at baseline, 1 h, and 24 h post-reperfusion. NETs markers (MPO-DNA, histone-DNA, citrullinated histone H3 [CitH3]) were measured in blood samples, and their associations with stroke severity (National Institutes of Health Stroke Scale [NIHSS] score at 24 h post-reperfusion), long-term functional outcome (modified Rankin Scale [mRS] score at 90-day), and final infarct size (5–7 days) were assessed in EVT and heparin + EVT–treated patients using logistic regression, linear regression, and Pearson’s correlation. Histone-DNA levels at 1 h post-heparin + EVT, but not EVT alone, were positively associated with final infarct size. Histone-DNA levels at 24 h post-heparin + EVT were negatively associated with infarct size mRS and NIHSS, while baseline CitH3 was positively correlated with NIHSS at 24 h post-EVT. Interaction analysis showed that the association between histone-DNA levels at 24 h and NIHSS at 24 h was different in the two treatment groups. No further associations were observed. At 1 h post-heparin + EVT, the histone-DNA levels were independently associated with larger infarct size, while at 24 h, histone-DNA linked to improved outcomes post-heparin + EVT and baseline-CitH3 to worse outcomes post-EVT, suggesting heparin may attenuate histone-DNA’s effect on outcome.

**Supplementary Information:**

The online version contains supplementary material available at 10.1007/s12975-025-01362-0.

## Introduction

Acute ischemic stroke (AIS), resulting from large-vessel occlusion in the anterior circulation, is commonly treated with endovascular thrombectomy (EVT) [1]. Despite the effectiveness of EVT, approximately half of the patients do not achieve functional independence within 3 months post-treatment [1]. Poor clinical outcomes may partially be caused by incomplete microvascular reperfusion (IMR) and thromboembolic complications during or after thrombectomy. IMR may arise from the in situ formation of microthrombi that cannot be reached by the stent retriever and from distal microvascular occlusion during EVT, potentially driven by neutrophil extracellular traps (NETs) [2–5], as a result, causing more brain tissue damage or large infarct size [6].

NETs are web-like structures composed of extracellular DNA, myeloperoxidase (MPO), histones, and antimicrobial proteins, released by neutrophils to trap and neutralize pathogens [7]. However, NETs also activate coagulation and inflammation, contributing to microthrombi formation and a prothrombotic state [8–10]. Several studies have found higher levels of NETs markers in plasma from patients with ischemic stroke compared to healthy individuals [11]. Also, histopathological studies have identified an abundant presence of NETs within thrombi retrieved from AIS patients, suggesting a significant role in stroke pathophysiology [12–14].

Much-studied NETs markers are citrullinated histone H3 (CitH3), MPO-DNA complexes, and histone-DNA complexes [15]. CitH3, produced during NETosis via peptidylarginine deiminase 4 (PAD4), drives chromatin decondensation, which is essential for NET formation. It promotes platelet activation and procoagulant microparticle release, and amplifies inflammation by activating immune cells [7, 8, 16–20]. High CitH3 levels have been associated with an increased 1-year mortality in patients with AIS [21]. MPO-DNA complexes, formed by the release of decondensed chromatin and MPO, enhance thrombin generation, stabilize thrombi by inhibiting fibrinolysis, and promote inflammation via neutrophil recruitment and cytokine release. Elevated MPO-DNA complex levels have been identified in patients with acute coronary syndrome and ischemic stroke [6, 22].

Histone-DNA complexes released during NETosis are cytotoxic and procoagulant. These complexes activate platelets, endothelial cells, and coagulation pathways while promoting inflammation through endothelial dysfunction and leukocyte recruitment [23, 24]. Elevated MPO-DNA and histone-DNA complexes are linked to cardiovascular events, thrombotic microangiopathies, and many inflammatory disorders, including sepsis, autoimmune diseases, and atherosclerosis [15, 25, 26]. Despite the involvement of NETs in prothrombotic states, limited clinical trials are available on the association of NETs with AIS outcomes. Many publications lack critical details, such as the timing of blood collection related to AIS onset or treatment form.

Interestingly, in vitro studies have demonstrated that heparin effectively degrades NETs [23, 24, 27, 28]. Beyond its anticoagulant properties, unfractionated heparin has been shown to dismantle NETs by disrupting the interactions between histones and extracellular DNA, leading to NET degradation [27, 28]. Heparin also neutralizes the cytotoxic and procoagulant effects of extracellular histones, thereby reducing thrombin generation, endothelial damage, and inflammatory cell recruitment [23, 24, 29]. In experimental models of thrombosis and inflammation, heparin administration decreased NET-mediated vascular occlusion and improved microvascular perfusion [27, 30]. These mechanistic insights support the hypothesis that periprocedural heparin during EVT might attenuate NETs-mediated thromboinflammation and reduce the risk of incomplete microvascular reperfusion. Based on this rationale, we investigated the association of NETs with clinical and radiological outcomes, such as short- (NIHSS scores at 24 h) and long-term (final infarct size at 5–7 days and mRS scores at 90 days) outcomes in AIS patients after EVT, and assessed how periprocedural heparin during EVT modulated NETs behavior and its association with clinical and radiological outcomes.

## Methods

### Patients 

We analyzed data from the Multicenter Randomized Clinical Trial of Endovascular Treatment for Acute Ischemic Stroke in the Netherlands (MR CLEAN-MED) trial with available blood samples. This open-label, multicenter, randomized clinical trial compared treatment with low-dose (5000 IU bolus followed by 500 IU/h for 6 h) unfractionated heparin plus EVT versus EVT alone. No patients in this substudy received IVT prior to EVT. Endovascular thrombectomy was performed with a Conformité Européenne–approved stent retriever. The study design was described previously [29]. Briefly, patient enrolment started on January 22, 2018, and was completed on February 4, 2021. The inclusion criteria were patients aged ≥ 18 years, ischemic stroke due to an intracranial large-vessel occlusion in the anterior circulation, admission within 6 h from symptom onset, a National Institutes of Health Stroke Scale (NIHSS) score ≥ 2, and no intracranial hemorrhage. The exclusion criteria were pre-stroke disability with a modified Rankin Scale score > 2, treatment with intravenous thrombolytics despite contraindications, contraindications for unfractionated heparin, use of heparin therapeutically, an international normalized ratio (INR) > 3.0, known hemorrhagic diathesis, and thrombocytopenia (platelet count < 90 × 10^9^ cells/L).

Patients underwent neurological assessments at several key time points. At baseline, they were evaluated using NIHSS score (ranging from 0 [indicating no symptoms] to 42 [representing the most severe deficits]), mRS score (ranging from 0 [indicating no disability] to 6 [indicating death]), and a non-contrast cerebral CT scan. The Alberta Stroke Program Early CT Score (ASPECTS) was used to quantify early ischemic damage on CT. The score ranges from 0, indicating extensive damage, to 10, reflecting no detectable ischemic damage. At the 24-h follow-up, patients were reassessed with NIHSS scores and either a cerebral CT or MRI scan (performed between 12 and 36 h after randomization). Between 5 and 7 days after the initial assessment, patients received a follow-up evaluation that included NIHSS scores and a non-contrast CT scan (if an MRI had not been conducted previously). Finally, a 90-day (± 14 days) follow-up was conducted, focusing on mRS scores to assess long-term outcomes.

The study protocols were in accordance with the Declaration of Helsinki and were approved by the Medical Ethics Committee of Erasmus MC University Medical Centre Rotterdam on 19–10–2017 (MEC-2017–366). Written informed consent was obtained from all participants, as detailed in the MR CLEAN-MED trial protocol [29].

### Blood Sampling

Blood samples were collected before treatment, 1 h, and 24 h after reperfusion treatment. Blood was drawn into sodium citrate tubes (final concentration 0.105 M) using a Vacutainer System (Beckton, Dickinson and Company, Plymouth, UK). Initially, the blood was centrifuged at 2500 g for 10 min at room temperature (RT). Subsequently, plasma was further centrifuged at 21,000 g for 10 min at room temperature (RT) and stored in aliquots at − 80 °C until analysis. Analysis included MPO-DNA complexes, histone-DNA complexes, citrullinated H3 from NETs, C-reactive protein (CRP [an indicator of inflammatory response]), and APTT and anti-Xa levels (indicators of the anticoagulant effect of heparin).

All the NETs markers were measured with the ELISA technique. We quantitatively measured histone-DNA complexes levels in plasma by using a commercial human cell death ELISA kit (Cell Death Detection ELISAPLUS, Cat. No. 11 920 685 001; Roche Diagnostics GmbH, Sandhofer Strasse 116, 68,305 Mannheim, Germany), as well as CitH3 levels by the ELISA kit (Citrullinated Histone H3 (Clone 11D3), Cayman CHMICAL, Cat. No. 501620, 1180 E. Ellsworth Rd Ann Arbor, MI, USA). For MPO-DNA complexes [30, 31], we adapted the commercial human cell death ELISA kit (Cell Death Detection ELISAPLUS, Cat. No. 11 920 685 001; Roche Diagnostics GmbH, Sandhofer Strasse 116, 68,305 Mannheim, Germany). We employed an anti-MPO monoclonal antibody (clone 4A4, Isotype IgG2b, Sanbio B.V., #0400–002) as the capturing antibody. Plasma samples from patients were added in combination with peroxidase-labeled anti-DNA monoclonal antibody (Component No. 2 of the Cell Death Detection ELISA kit; Roche, #11–774-425–002) in the solution. Absorbance was measured at 450 nm wavelength using a Biotek Synergy HT plate reader, with a reference filter set to 490 nm. As a control, we induced NETosis in healthy volunteers by using Lymphoprep (Prod. No. 1114544, Serumwerk Bernburg AG, for Alere Technologies AS, Oslo, Norway) and stored as aliquots in fetal calf serum until the analysis of MPO-DNA complexes. The values were expressed in arbitrary units per microliter (AU/mL). C-reactive protein levels were determined in the serum of patients using Cardiac C-Reactive Protein (Latex) High Sensitive (hs-CRP) kits on a Roche/Hitachi cobas c 501 analyzer (Cat. 04628918 190, Roche Diagnostics GmbH, Sandhofer Strasse 116, D-68305 Mannheim). Anti-Xa levels were determined in plasma using a chromogenic substrate (HemosIL Liquid Anti-Xa Assay, Instrumentation Laboratory Company) on the Sysmex CS5100. Activated partial thromboplastin time (APTT) was measured in plasma with Actin FS (SIEMENS) on the Sysmex CS5100. Reference ranges for plasma markers were defined using healthy individuals.

### Outcome Parameters and Statistical Analysis

The primary outcome of this study was final infarct volume, measured on non-contrast CT obtained 5–7 days after presentation. Secondary outcomes were neurological deficit at 24 h, assessed using the NIHSS, and functional outcome at 90 days, evaluated with the mRS score.

We presented baseline characteristics of all included patients and of patients stratified by randomization for receiving periprocedural heparin or not. NETs markers were analyzed in the available plasma from all patients before and at 1 h and 24 h after reperfusion to assess their association with outcomes after EVT and the impact of heparin on these associations. Normally distributed data are shown as mean and standard deviation (SD), non-normally distributed data as median and 25th–75th percentiles, and categorical data as number and percentage. Groups were compared using the chi-square test for categorical variables, and the Mann–Whitney *U*-test for non-normally distributed continuous variables. For multiple group comparisons, a Bonferroni adjustment was used. Pearson’s correlation coefficient was employed to evaluate the linear association between NETs markers and clinical or radiological outcomes. To investigate the association between NETs markers (independent variables) and final infarct volume, NIHSS scores, and ASPECTS (dependent variables), linear regression analysis was used. The linear regression findings were expressed as beta (β) coefficients and 95% confidence interval (CI). The data for infarct size and NIHSS scores were log-transformed due to their non-normal distribution. Logistic regression was performed to determine the association between NETs markers (independent variables) and mRS scores (dependent variable). Logistic regression results were expressed as odds ratio (OR) and 95% confidence interval (CI). Both linear and logistic regression analyses were adjusted for age, sex, weight, diabetes mellitus, atrial fibrillation, collateral status, baseline mRS scores, baseline NIHSS scores, baseline ASPECTS, and time from onset to randomization. Interaction analyses were conducted to determine whether heparin modified the association between NETs markers and clinical or radiological outcomes. Both adjusted and unadjusted models were used, applying logistic regression for binary outcomes and linear regression for continuous outcomes. Statistical significance was considered if the *p*-value was ≤ 0.05. Statistical analyses were performed using IBM SPSS version 25 (IBM Corp.). Graphics were created using GraphPad Prism version 8.2.1 (GraphPad Software, San Diego, CA, USA).

## Results

### Patient’s Baseline Characteristics

Patient and stroke characteristics are presented in Tables 1 and 2. The MR CLEAN-MED trial included 628 patients, of whom 297 were randomized to receive either heparin plus EVT or EVT alone. Blood samples were available from 198 of these 297 patients, forming the subset analyzed in this study [29]. Among the 198 patients with blood samples, 104 received unfractionated heparin in addition to EVT, and 94 received EVT alone. In the heparin plus EVT group, blood samples were collected from 74 patients before treatment, 75 patients 1 h after, and 46 patients 24 h after treatment. In the EVT-alone group, blood samples were collected from 70 patients before treatment, 65 patients 1 h after, and 46 patients 24 h after treatment. Pre-stroke functional status, as measured by the modified Rankin Scale (mRS), was comparable between the groups. In the heparin plus EVT group, 63.8% of patients had no symptoms (mRS 0), 23.8% had symptoms without disability (mRS 1), 11.4% had slight disability (mRS 2), and 1.0% had moderate disability (mRS ≥ 3). In the EVT-alone group, these proportions were 72.3%, 13.8%, 6.4%, and 7.4%, respectively.

**Table 1:** Patients’ baseline characteristics

Demographics	Low-dose heparin + EVT* (*n* = 104)	EVT alone (*n* = 94)	Total patients (*n* = 297)
Age, years	72.0 (62.0–79.5)	72.5 (64.8, 83.0)	73.0 (63.0–81.0)
Male, *n* (%)	58 (55.2)	55 (58.5)	166 (55.9)
Weight, kg	80.0 (71.5–90.5)	79.0 (70.0, 86.5)	79.0 (70.0–90.0)
**Medical history**			
Diabetes mellitus, *n* (%)	17 (16.2)	12 (12.8)	41 (13.8)
Atrial fibrillation, *n* (%)	26 (24.8)	27 (28.7)	72 (24.2)
**Medication**			
Vitamin K antagonists, *n* (%)	7 (6.7)	11 (11.7)	25 (7.7)
DOAC, *n* (%)	12 (11.4)	12 (12.8)	31 (10.4)
Baseline NIHSS score#	17.0 (10.0–21.0)	13 (7.5, 19.0)	15.0 (9.0–19.0)
Systolic blood pressure, mmHg	150 (137–162)	149 (130, 161)	150 (135–164)
Glucose level, mmol/L	6.7 (5.9–7.7)	6.9 (6.1, 7.9)	6.8 (6.0–7.7)
**Stroke history, *n* (%)**			
Ischemic stroke	18 (17.1)	20 (21.3)	54 (18.2)

Abbreviations: *EVT* endovascular treatment

*Number of patients treated with low-dose unfractionated heparin plus EVT from whom blood samples were collected

Number of patients treated with EVT alone from whom blood samples were collected

The total number of patients included for heparin plus EVT and EVT alone in the MR CLEAN-MED trial

^#^Scores on the National Institutes of Health Stroke Scale (NIHSS) range from 0 to 42, with higher scores indicating a more severe neurologic deficit

Normally distributed data are shown as mean and SD, and non-normally distributed data as median and 25th–75th percentiles. Categorical data are shown as number and percentage

**Table 2:** Stroke event and procedural characteristics

Characteristics	Low-dose heparin + EVT* (*n* = 104)	EVT alone (*n* = 94)	Total patients (*n* = 297)
**Location of intracranial occlusion, *n* (%)**			
Intracranial ICA	9 (8.6)	9 (9.7)	24 (8.1)
Terminal ICA	24 (22.9)	12 (12.9)	55 (18.6)
M1	53 (50.5)	50 (53.8)	156 (52.7)
M2	18 (17.1)	22 (23.7)	59 (19.9)
None	1 (1.0)	0	2 (0.7)
**Collateral score, *n* (%)**			
Poor collateral (0–1)	34 (32.7)	26 (28.0)	97 (33.0)
Good collateral (2–3)	70 (67.3)	67 (72.0)	197 (67.0)
**Duration, min**			
From stroke onset to randomization	159 (118–208)	167 (131–214)	160 (126–208)
From stroke onset to start of alteplase	72 (58–128)	78 (60–130)	80 (60–127)
From stroke onset to groin puncture	170 (140–225)	190(144–243)	175 (142–225)
From door to needle	218 (180–278)	225 (186–272)	220 (180–274)
From needle to groin puncture	45 (30–68)	49 (30–66)	45 (30–70)
From door to groin puncture	14:23:00 (10:45:00–18:45:00)	15:28:00 (12:15:00–20:00:00)	14:30:30 (11:07:30–18:48:45)
**New stroke after treatment, *n* (%)**			
Stroke progression	15 (14.3)	8 (8.5)	34 (11.4)
New ischemic stroke	4(3.8)	5 (5.3)	17 (5.7)
sICH	6 (5.7)	3 (3.2)	20 (6.7)

Abbreviations: *EVT* endovascular thrombectomy, *ICA* internal carotid artery, *M1* first segment of the middle cerebral artery, *M2* second segment of the middle cerebral artery. >The location of the intracranial occlusion was scored by the core laboratory. Patients who had an isolated occlusion of the extracranial ICA and thus were scored as having no intracranial occlusion *sICH* Symptomatic intracranial hemorrhage

^*^Number of patients treated with low-dose unfractionated heparin plus EVT from whom blood samples were collected

Number of patients treated with EVT alone from whom blood samples were collected

The total number of patients included for heparin plus EVT and EVT alone in the MR CLEAN-MED trial

Normally distributed data are shown as mean and SD, and non-normally distributed data as median and 25th–75th percentiles. Categorical data are shown as number and percentage

Overall, patient and stroke characteristics were similar between the two study groups and consistent with the overall MR CLEAN-MED population (Tables 1, 1).

### Effect of Heparin Plus EVT Treatment on Levels of NETs Markers

**Fig. 1:**
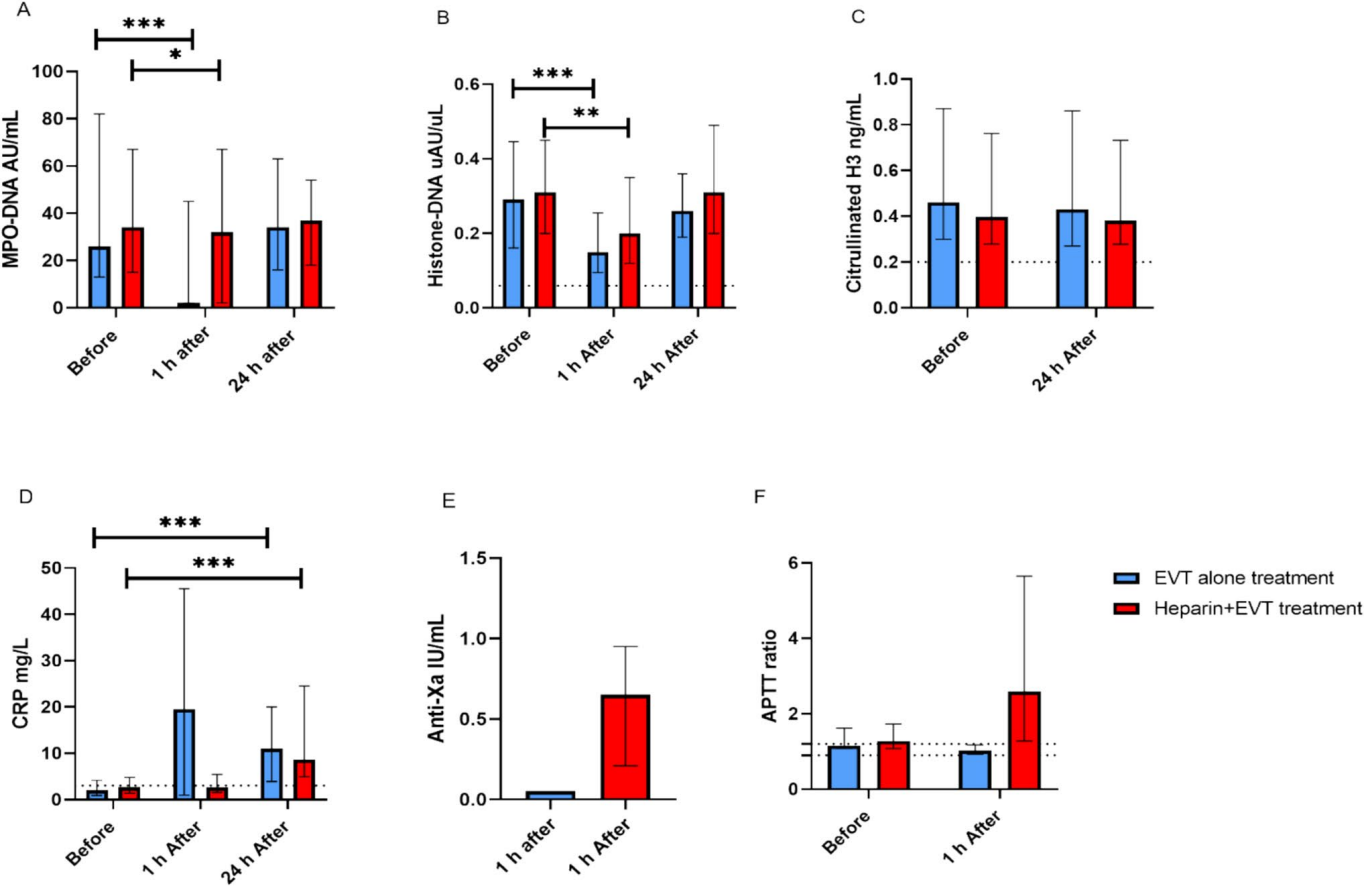
Effect of periprocedural heparin during EVT or EVT alone on levels of markers (NETs (MPO-DNA, histone-DNA, citrullinated H3 (**A**–**C**)), CRP (**D**), anti-Xa (**E**), and APTT ratio (**F**)) before and/or 1 h and 24 h after reperfusion. Levels of markers before EVT, 1 h after, and 24 h after EVT alone are shown as blue bars; and levels of markers before, 1 h after, and 24 h after heparin + EVT are indicated in red bars. Upper and lower limits of reference are indicated with black dashed lines. Concentration values of MPO-DNA and CRP are presented on a logarithmic scale to accommodate the wide range of data and to improve visualization of lower reference values. Abbreviations: EVT, endovascular thrombectomy treatment; NETs, neutrophil extracellular traps; CRP, C-reactive protein; APTT, activated partial thromboplastin time; EVT, thrombectomy. Data are reported as median and 25th–75th percentiles (not-normally distributed), *p ≤ 0.05; **p ≤ 0.01; ***p ≤ 0.001

Figure 1A–C depicts NETs levels measured at baseline, 1 h, and 24 h post-reperfusion in both groups, with detailed values provided in Table S1. In both treatment groups, MPO-DNA levels (Fig. 1A) were elevated at all three time points compared to the reference levels. Only a weak reduction in MPO-DNA levels by heparin was observed at 1 h after reperfusion (Fig. 1A), while MPO-DNA levels in the EVT-alone group had decreased more. At 1 h, levels in both groups were significantly lower compared to baseline, but returned to baseline levels at 24 h post-treatments (Fig. 1A). Histone-DNA levels (Fig. 1B) were significantly elevated prior to treatment with either heparin plus EVT or EVT alone compared to reference levels. At 1 h post-treatment, levels were decreased in both groups; however, the reduction was more pronounced in patients treated with EVT alone compared to those receiving heparin plus EVT. At 24 h post-treatment, histone-DNA levels had returned to baseline in both treatment groups. Also, CitH3 levels (Fig. 1C) were elevated prior to the interventions compared to reference levels, and they remained unchanged at 24 h following reperfusion treatments.

Changes in the levels of all three NETs markers over time were consistent before and after both treatments. In both treatment groups, baseline NETs biomarker levels were positively correlated with each other and with the levels over time, either at 1 h and/or 24 h after reperfusion (Table S2). However, baseline CitH3 levels after heparin treatment showed no significant correlation with their levels at 24 h, while a significant correlation was observed in the EVT-alone group (Table S2). All NETs markers except CitH3 decreased significantly more following EVT-alone treatment compared to levels observed after heparin plus EVT treatment (Table S2).

CRP levels significantly increased at 1 h post-EVT alone, which correlated with the decrease in MPO-DNA levels (Fig. 1A, D, Table S2). Furthermore, in both treatment groups, CRP levels at 24 h post-treatment were increased compared to levels before the treatment; however, the levels were more increased after EVT-alone treatment. We observed a correlation between high CitH3 levels before treatment and elevated CRP after EVT-alone treatment (Table S2).

We confirmed that patients in the heparin plus EVT group did receive their heparin by APTT and anti-Xa levels (Fig. 1E, F).

### Association of NETs Markers with Clinical and Radiological Outcomes After Treatments

Levels of NETs markers on admission were not significantly associated with baseline stroke severity (NIHSS) or early ischemic brain damage (ASPECTS) after adjusting for confounders (Supplementary Tables S3, S4, Fig S1).

In the heparin plus EVT treatment group, histone-DNA levels at 1 h post-reperfusion were positively associated with final infarct size at 5–7 days, after adjusting for confounders (*β* = 0.46 [95% CI 0.03, 0.89], *p*-value = 0.04) (Tables 3, S5). Conversely, at 24 h post-heparin plus EVT treatment, histone-DNA levels were negatively associated with reduced final infarct volume (Tables 3, S5, S6). These associations were not observed in the EVT-alone treatment group. Additionally, histone-DNA levels at 24 h post-heparin plus EVT were negatively correlated with mRS scores and NIHSS scores (*r* = − 0.36, *p* = 0.02; *r* = − 0.42, *p* = 0.004, respectively) (Table S6). Interaction analysis showed that heparin treatment significantly modified the association between histone-DNA levels at 24 h and NIHSS scores at 24 h, without and with adjustments for covariates (*β* = − 1.14, 95% CI [− 1.94, − 0.34]; *β* = − 0.98, 95% CI [− 1.80, − 0.17], *p* = 0.02, respectively).

**Table 3:** Association of NETs markers before, 1 h, and 24 h after heparin plus EVT or EVT-alone treatment with NIHSS scores at 24 h, final infarct size at 5–7 days, and mRS scores at 90 days after treatment in AIS patients

Markers	NIHSS scores		Final infarct size		mRS score (unfavorable ≥ 3)	
	***β* (95% CI)**	***p*-value**	***β* (95% CI)**	***p*-value**	**ODDs (95% CI)**	***p*-value**
**NETs**						
**MPO-DNA complexes (AU/µL)**						
Before EVT + Hep treatment	0.24 (− 0.84, 1.13), 62	0.66	0.12 (− 0.43, 0.66), 58	0.66	5.11 (0.63, 41.53), 65	0.13
1 h after EVT + Hep	0.25 (− 0.67, 1.17), 62	0.59	− 0.22 (− 0.59, 0.14), 58	0.23	0.92 (0.25, 3.45), 65	0.90
24 h after EVT + Hep	0.21 (− 1.48, 1.91), 39	0.80	− 0.05 (− 0.74, 0.65), 35	0.88	1.66 (0.16, 17.03), 39	0.67
Before EVT-alone treatment	− 0.68 (− 1.67, 0.32), 58	0.18	− 0.24 (− 0.75, 0.28), 51	0.36	0.43 (0.08, 2.35), 62	0.33
1 h after EVT alone	− 0.56 (− 1.39, 0.27), 59	0.18	− 0.45 (− 0.92, 0.02), 50	0.06	0.82 (0.14, 4.66), 61	0.82
24 h after EVT alone	− 0.34 (− 1.45, 0.76), 39	0.53	− 0.16 (− 0.80, 0.48), 32	0.61	1.07 (0.98, 1.16), 38	0.12
**Histone-DNA complexes (ng/mL)**						
Before EVT + Hep treatment	0.01 (− 0.97, 0.99), 62	0.99	0.18 (− 0.33, 0.70), 58	0.48	2.76 (0.51, 14.79), 65	0.24
1 h after EVT + Hep	0.84 (− 0.31, 2.00), 62	0.13	**0.46 (0.03, 0.89), 58**	**0.04**	4.79 (0.81, 28.28), 65	0.08
24 h after EVT + Hep	− 1.34 (− 2.76, 0.08), 38	0.06	− 0.27 (− 0.33, 1.29), 34	0.23	0.01 (0.00, 1.39), 38	0.08
Before EVT-alone treatment	− 0.13 (− 1.01, 0.76), 58	0.78	− 0.22 (− 0.69, 0.26), 51	0.36	0.35 (0.05, 2.37), 62	0.28
1 h after EVT alone	− 0.38 (− 1.32, 0.58), 59	0.43	− 0.07 (− 0.67, 0.53), 50	0.82	0.25 (0.03, 2.25), 61	0.22
24 h after EVT alone	0.75 (− 0.39, 1.89), 38	0.19	0.17 (− 0.74, 1.08), 31	0.69	1.18 (0.07, 20.25), 40	0.23
**CitH3 (ng/L)**						
Before EVT + Hep treatment	− 0.05 (− 0.17, 0.07), 61	0.41	− 0.01 (− 0.07, 0.05), 56	0.81	0.84 (0.25, 2.76), 62	0.77
24 h after EVT + Hep	− 0.02 (− 0.16, 0.13), 39	0.80	− 0.01 (− 0.07, 0.05), 35	0.73	0.95 (0.59, 1.54), 39	0.84
Before EVT alone	0.17 (− 0.57, 0.91), 58	0.65	0.01 (− 0.39, 0.42), 51	0.95	0.49 (0.12, 1.99), 62	0.32
24 h after EVT alone	− 0.42 (− 1.61, 0.77), 39	0.48	0.39 (− 0.40, 1.18), 32	0.31	0.09 (0.003, 2.68), 41	0.67

Adjusted for the diabetes mellitus, atrial fibrillation, age, sex, weight, baseline mRS score, collateral status, time from onset to randomization, baseline ASPECTS, baseline NIHSS score. Linear or logistic regression was used to investigate the association between NETs, inflammation, and coagulation biomarkers and NIHSS scores at 24 h post-reperfusion, final infarct size at 5–7 days, and mRS scores at 90 days post-reperfusion. Abbreviations: MPO-DNA complexes, Myeloperoxidase DNA complexes; CitH3, Citrullinated histone H3; EVT, Endovascular thrombectomy; EVT+ Hep, Endovascular thrombectomy plus heparin; NIHSS score, National Institutes of Health Stroke Scale score; mRS score, Modified Rankin Scale score; ASPECTS, Alberta Stroke Program Early CT Score

In the EVT-alone treatment group, regression analysis showed no association between NETs markers (at all time points) and clinical or radiological outcomes (Tables 3, S5, S7, S8). However, a positive correlation was observed between increased baseline CitH3 levels and severe NIHSS scores at 24 h post-reperfusion (*r* = 0.28, *p* = 0.03), but not with infarct size and mRS scores. This correlation was not observed in patients treated with heparin plus EVT (Table S6).

## Discussion

The main findings of this study are the positive association between NETs marker histone-DNA complex levels 1 h after treatment with final infarct size in the group EVT plus heparin, an association absent in the EVT-alone group. Interaction analysis showed that heparin treatment significantly modified the association between histone-DNA levels at 24 h and stroke severity. The effect estimates of histone-DNA complexes at 24 h after heparin plus EVT and their association with clinical and radiological outcomes showed a similar trend, although not statistically significant. Also, exploratory correlation analyses revealed some patterns of interest. Correlation analysis showed negative associations between histone-DNA levels 24 h post-heparin plus EVT and final infarct volume, long-term functional outcome, and stroke severity. In contrast, within the EVT-alone treatment group, elevated baseline CitH3 levels were positively correlated with stroke severity at 24 h post-reperfusion. MPO-DNA and histone-DNA levels were decreased 1 h after treatment compared to baseline but returned to baseline levels at 24 h after treatment of both heparin plus EVT or EVT alone. No clear associations were seen for the MPO-DNA complex or the citrullinated H3 levels with clinical and radiological outcomes.

We found that high histone-DNA complexes levels at 1 h after EVT were positively associated with our primary outcome, final infarct size, in the heparin plus EVT treatment group, but not in the EVT-alone group. Large infarct size is a known surrogate for poor neurological outcome, and understanding its early drivers is essential. No other studies measured NETs markers in plasma of AIS patients undergoing EVT with or without heparin. Our finding suggests that early NET formation contributes to brain tissue damage after EVT. Endovascular thrombectomy procedures can cause microvascular obstruction by stimulating neutrophil NETs release [2–5]; excessive NETs activity may therefore promote secondary ischemic injury and infarct growth. The observed association between histone-DNA levels and infarct size at 1 h after EVT in the heparin plus EVT group likely reflects an acute surge in cell death and NET formation triggered by ischemia–reperfusion injury and mechanical thrombectomy, rather than a pro-NET effect of heparin itself, as the specific NETs markers show a similar trend. However, an alternative explanation is that heparin may have released NET components trapped in the brain’s microvasculature into the systemic circulation, thereby revealing the extent of downstream microvascular thrombosis, a process associated with poor outcomes in preclinical models. In contrast, in patients treated with EVT alone, NETs may remain sequestered within the microvasculature, potentially masking the underlying microvascular burden. Although low-dose heparin may modulate NETs-related processes by releasing histones and destabilizing the extracellular DNA structure [28], its effect appears limited during the first hour, likely due to subtherapeutic concentrations insufficient to neutralize circulating histones. After 24 h, in the heparin plus EVT group where patients received low-dose heparin during the first 6 h of EVT, a modest beneficial effect emerges but remains inadequate to markedly reduce NET burden or mitigate brain tissue damage. Histones are known to drive procoagulant and inflammatory responses, exacerbating tissue damage [23, 28], and the low dose of heparin appears insufficient to fully degrade these extracellular cytotoxic components. Alternatively, heparin may be more effective at destabilizing circulating NETs, where histone-DNA complexes are freely accessible, than intravascular NETs embedded within thrombi. Although direct evidence comparing the susceptibility of circulating and intravascular NETs to heparin is currently lacking, this difference remains biologically plausible [9]. The inflammatory role of NETs, as suggested by our exploratory findings, is supported by a positive correlation between CRP levels and NETs markers at early time point after heparin plus EVT. We interpret this as a suggestion that CRP may reflect a NETs-driven systemic inflammatory, rather than being a direct mediator. This is consistent with previous findings that NETs can stimulate hepatic CRP production via proinflammatory pathways [2, 3], and with our observation that CRP and CitH3 levels remain correlated at 24 h in the EVT-alone group but not in the heparin-treated group, suggesting that heparin may modulate NETs-induced inflammation over time. This dynamic implies that the timing of NETs degradation is crucial; early NETs clearance may reduce their damaging effects and improve outcomes. Our exploratory data also support that heparin may modulate inflammation over time by dysregulating CitH3, as evidenced by the higher CitH3 and CRP levels at 24 h in the EVT-alone group, along with a correlation between these markers, which is absent in the heparin plus EVT group.

The temporal dynamics of NETs formation warrant further consideration. CitH3 is typically regarded as an early marker of PAD4-mediated NETosis, while extracellular MPO-DNA complexes may reflect later-stage NET decondensation. In our cohort, MPO-DNA and histone-DNA levels declined shortly after treatment but returned to baseline at 24 h, suggesting involvement of overlapping NETosis pathways [26, 32, 33]. It is plausible that both vital NETosis, characterized by NET release without neutrophil death, and suicidal NETosis, involving complete neutrophil lysis, contributed to this circulating NETs profile [34]. Furthermore, the kinetics of NET clearance may have influenced our findings. Histones and CitH3 are rapidly degraded by proteases and DNases, while MPO-DNA complexes might be relatively more stable [26, 35], leading to differential detection over time.

Our findings align with growing experimental and clinical evidence implicating NETs and neutrophil activation in ischemic stroke. Denorme et al. (2022) demonstrated that NETs form early after cerebral ischemia and actively promote thrombo-inflammatory brain injury through microvascular obstruction and neuronal damage [36]. Similarly, the NEUTROSTROKE study showed that elevated neutrophil activation markers after thrombectomy were associated with poor outcomes, while prior intravenous thrombolysis attenuated this effect [37]. These recent studies underscore the importance of NET-mediated thromboinflammation in AIS and provide critical context for interpreting our findings. Interestingly, in our study, patients treated with EVT alone show no association between NETs markers and infarct size at an early stage, whereas those treated with heparin do. However, exploratory correlation analysis suggests a link between NETs and inflammation following EVT alone with stroke severity and long-term functional outcomes, as demonstrated by the positive correlation of CitH3 and CRP with NIHSS scores at 24 h and mRS scores at 90 days. In contrast, correlation analysis also reveals a significant link between histone-DNA levels and improved clinical and radiological outcomes 24 h after heparin treatment. Similarly, regression analysis suggests a trend toward this association. Interaction analysis further supported these findings, showing that heparin may modulate the relationship between histone-DNA levels at 24 h and stroke severity, highlighting its potential role in post-stroke outcomes, although these findings are exploratory and require validation.

The MR CLEAN-MED trial reported that periprocedural intravenous unfractionated heparin during EVT showed no evidence for a beneficial effect on functional outcome and was even associated with an increased risk of symptomatic intracranial hemorrhage with the higher doses heparin [29]. In our study, we only analyzed patients who received a low dose of heparin (e.g., 5000 IU followed by 500 IU/h for 6 h). A method to improve NETs degradation and outcomes may be to also administer DNase, an agent that also dismantles NETs [38, 39]. Ex vivo studies have shown that DNase I significantly enhances thrombolysis by degrading extracellular DNA, the structural backbone of NETs (e.g., histone-DNA complexes) [39], and reduces infarct volume [6]. Although preclinical data support this combination of DNase and low-dose heparin, clinical trials are needed to evaluate their efficacy and safety in AIS patients undergoing EVT and also to validate our findings by assessing the association of NETs at different time points with clinical and radiological outcomes in patients undergoing EVT with or without heparin.

## Strength and Limitations

The strength of our study was that we prospectively and consecutively collected patient samples in a well-designed randomized clinical trial, and blood samples were obtained before treatment, as well as 1 h and 24 h after treatment. Additionally, we analyzed various NETs markers.

However, there are several limitations: First, blood samples were not collected at all three time points for every patient due to logistic reasons. However, the characteristics of our subgroup were comparable to those of the overall group, indicating that the present analysis is representative of the total study population. Second, the sample size per group and time point, particularly at 24 h, was limited, which may affect statistical power and the reliability of observed associations. Moreover, the number of statistical tests performed was relatively high in relation to the sample size, increasing the risk of false-positive findings due to alpha error inflation, as well as false negatives due to limited power. Third, we were unable to include patients treated with high-dose heparin during EVT in the analysis due to the small sample size. Fourth, despite randomization in the MR CLEAN-MED trial, a baseline imbalance in stroke severity was present between the groups that received EVT plus heparin versus EVT alone, which may have introduced residual confounding despite adjustment. Finally, given the exploratory nature of our analyses and the absence of a direct benefit of heparin on clinical outcomes in the main trial, our findings should be interpreted as hypothesis-generating.

## Conclusion

Histone-DNA complex levels 1 h after EVT were positively associated with final infarct size in the heparin plus EVT group, after adjusting for confounders, but not in the EVT-alone group. However, at 24 h post-heparin plus EVT, this association shifted to a negative trend with infarct size. Additionally, heparin appeared to improve stroke severity by modulating the relationship between histone-DNA complex levels at 24 h and NIHSS scores, suggesting a potential distinct interaction between heparin and NETs over time. Also, exploratory correlation analysis revealed that histone-DNA levels at 24 h post-heparin plus EVT negatively correlated with reduced infarct size and improved clinical outcomes, while baseline CitH3 levels were positively associated with short-term stroke severity 24 h post-EVT alone. Although low-dose heparin may influence NETs-related processes, it appears insufficient to neutralize histones. These findings suggest that strategies aimed at enhancing NETs degradation, such as combining heparin with DNase, warrant further investigation through randomized clinical trials to optimize early outcomes in AIS patients undergoing EVT.

## Supplementary Information

Below is the link to the electronic supplementary material.

Supplementary file1 (DOCX 148 KB)

## Data Availability

No datasets were generated or analysed during the current study.
