# Supplementary material for: Association of NETs Markers with Clinical and Radiological Outcomes in Patients with Acute Ischemic Stroke Undergoing Thrombectomy: Does Heparin Treatment Modify This?
Source: Transl Stroke Res. 2025 Jun 26;16(6):1985–95. doi: 10.1007/s12975-025-01362-0 (PMC12596317; doi:10.1007/s12975-025-01362-0)
Supplement: Supplementary file 1 — Supplementary file1 (DOCX 148 KB) [file 12975_2025_1362_MOESM1_ESM.docx]

**Supplemental Material On ‘’**Association of NETS and inflammation biomarkers with clinical and radiological outcomes in patients with acute ischemic stroke undergoing thrombectomy: does heparin treatment modify this?”

Aarazo Barakzie^1^, Wouter van der Steen^2,3^, A.J. Gerard Jansen^1^, Bob Roozenbeek^2,3^, Samantha J. Donkel^1^, Aad van der Lugt^4^, Hester Lingsma^3^, Diederik W.J. Dippel^2,3^, Hugo ten Cate^5,6^, Moniek P. M. de Maat^1^, on behalf of the CONTRAST consortium.

^1^Department of Hematology, Erasmus MC Cardiovascular Institute, University Medical Center Rotterdam, Rotterdam, The Netherlands.

^2^Department of Neurology, Erasmus MC, University Medical Center Rotterdam, Rotterdam, The Netherlands.

^3^Department of Public Health, Erasmus MC, University Medical Center Rotterdam, Rotterdam, The Netherlands.

^4^Department of Radiology and Nuclear Medicine, Erasmus MC, University Medical Center Rotterdam, Rotterdam, The Netherlands.

^5^Department of Biochemistry, CARIM School for Cardiovascular Diseases, Maastricht University Medical Centre, The Netherlands.

^6^Thrombosis Expertise Center and Department of Internal Medicine, Maastricht University Medical Centre, The Netherlands.

Content

**SUPPLEMENTARY** 3

**SUPPLEMENTARY Table S1:** levels of hemostatic biomarkers in AIS patients and the effect of heparin during EVT or EVT alone on these biomarkers at different time points of reperfusion..........3

**SUPPLEMENTARY Table S2:** Correlation between NETs biomarkers.........................….......................5

**SUPPLEMENTARY Table S3:** Association of NETs, inflammation, and coagulation biomarkers before heparin plus EVT or EVT alone treatment with baseline NIHSS scores.…………………....…......................7

**SUPPLEMENTARY Table S4:** Association of NETs, inflammation, and coagulation biomarkers before heparin plus EVT or EVT alone treatment with baseline ASPECTS scores............................................8

**SUPPLEMENTARY Table S5:** Association of NETs, inflammation, and coagulation biomarkers before, 1 h, and 24 h after heparin plus EVT or EVT alone treatment with final infarct size at 7 days after treatment in AIS patients....................................................................................................................9

**SUPPLEMENTARY Table S6:** Correlation between biomarkers and outcomes……..................…........11

**SUPPLEMENTARY Table S7:** Association of NETs, inflammation, and coagulation biomarkers before, 1 h, and 24 h after heparin plus EVT or EVT alone treatment with NIHSS scores at 24h after treatment in AIS patients....................................................................................................................12

**SUPPLEMENTARY Table S8:** Association of NETs, inflammation, and coagulation biomarkers before, 1 h, and 24 h after heparin plus EVT or EVT alone treatment with unfavorable long-term functional outcome (3-6 mRS) at 90 days after treatment in AIS patients..........................................................14

**SUPPLEMENTARY Figure S1:** Association of NETs markers levels before heparin plus EVT or EVT alone treatment with stroke severity (NIHSS scores) and brain damage (ASPECTS) on admission....16

**Acknowledgement:** 17

# **Supplemental Tables**

| **SUPPLEMENTARY Table S1:** levels of hemostatic biomarkers in AIS patients and the effect of heparin during EVT or EVT alone on these biomarkers at different time points of reperfusion: | | | | | | |
| --- | --- | --- | --- | --- | --- | --- |
| **Biomarkers** | **Reference** | **Blood Draw Time Points** | | | **P-value** | |
|  |  | **Before treatment** | **1h after treatment** | **24h after treatment** | **Before treatment vs 1h after treatment** | **Before treatment vs 24h after treatment** |
| **MPO-DNA (AU/uL)** | | | | | | |
| Heparin+EVT | 0.00005 | 0.03 [0.02-0.07] | 0.032 [0.002-0.07] | 0.037 [0.02-0.05] | **0.04** | **0.52** |
| EVT alone |  | 0.03 [0.013-0.082] | 0.002 [0.002-0.04] | 0.03 [0.02-0.06] | **<0.001** | 0.90 |
| **Histone-DNA (ng/mL)** | | | | | | |
| Heparin+EVT | 0.06 | 0.31 [0.20-0.45] | 0.20 [0.12-0.35] | 0.31 [0.20-0.49] | **0.002** | **0.47** |
| EVT alone |  | 0.29 [0.16-0.45] | 0.15 [0.10-0.26] | 0.26 [0.19- 0.36] | **<0.001** | 0.65 |
| **CitH3 (ng/mL )** | | | | | | |
| Heparin+EVT | 0.20 [0.0-0.70] | 0.46 [0.30-0.87] | NA | 0.43 [0.27-0.86] | NA | 1.00 |
| EVT alone |  | 0.40 [0.28-0.76] | NA | 0.38 [0.28-0.73] | NA | 0.80 |
| **CRP (mg/L)** | | | | | | |
| Heparin+EVT | <3 | 2.70 [1.40-4.78] | 2.65 [1.50-5.45] | 8.70 [4.95-24.50] | 0.88 | **<0.001** |
| EVT alone |  | 2.00 [0.90, 4.15] | 19.50 [0.93-45.50] | 11.00 [3.90, 20.0 | 0.79 | **<0.001** |
| **APTT ratio** | | | | | | |
| Heparin+EVT | 0.8-1.2 | 1.28 [1.08-1.73] | 2.59 [1.28-5.65] | NA | **<0.001** | NA |
| EVT alone |  | 1.16 [1.08-1.62] | 1.02 [0.93, 1.18] | NA | **0.03** | NA |
| **Anti_Xa (IU/mL)** | | | | | | |
| Heparin+EVT | 0.3-0.7 | NA | 0.64 [0.21-0.95] | NA | NA | NA |
| EVT alone |  | NA | 0.05 [0.05-0.05] | NA | NA | NA |
| Data are shown as median and 25th-75th percentiles, for comparing this not normally distributed data we used the Mann-Whitney U test. Abbreviation: Myeloperoxidase DNA complexes = MPO-DNA , Citrullinated histone H3 = CitH3, C-reactive protein = CRP, Activated partial thromboplastin time = APTT, Not applicable = NA, Endovascular thrombectomy alone = EVT alone treatment, Endovascular thrombectomy + heparin treatment = EVT plus heparin treatment. | | | | | | |

| **SUPPLEMENTARY Table S2: linear association between NETs markers, using Pearson's correlation coefficient.** | | | | | | | | | | | |
| --- | --- | --- | --- | --- | --- | --- | --- | --- | --- | --- | --- |
| **Blood collection time point** | **Biomarkers** |  | | | | | | | | | |
|  |  | **1 h after treatment** | **24 h After treatment** | **Before treatment** | **1 h after treatment** | **24 h After treatment** | **Before treatment** | **24 h After treatment** | **Before treatment** | **1 h after treatment** | **24 h After treatment** |
|  |  |  | | **Histone-DNA** | | | **CitH3** | | **CRP** | | |
| **Heparin+EVT** | | | | | | | | | | | |
| **Before treatment** | **MPO-DNA** | 0.68 | **0.94**** | 0.41 | 0.23 | **0.50*** | 0.03 | 0.15 | 0.19 | **0.27*** | 0.05 |
| **1 h after** |  |  | **0.67**** | 0.17 | **0.30**** | **0.57**** | 0.14 | -0.13 | **0.30*** | 0.17 | 0.20 |
| **24 h after treatment** |  |  |  | 0.19 | **0.41*** | 0.34* | 0.31 | 0.04 | 0.32 | 0.31 | 0.06 |
| **Before treatment** | **Histone-DNA** |  |  |  | 0.21 | **0.54**** | **0.27*** | 0.21 | -0.08 | -0.03 | 0.09 |
| **1 h after** |  |  |  |  |  | 0.10 | 0.01 | 0.11 | 0.11 | 0.03 | -0.14 |
| **24 h after treatment** |  |  |  |  |  |  | 0.28 | **0.32*** | 0.25 | 0.33 | 0.16 |
| **Before treatment** | **CitH3** |  |  |  |  |  |  | 0.24 | 0.01 | 0.02 | 0.28 |
| **24 h after treatment** |  |  |  |  |  |  |  |  | 0.14 | 0.001 | -0.10 |
| **Before treatment** | **CRP** |  |  |  |  |  |  |  |  | **0.93**** | 0.47 |
| **1 h after** |  |  |  |  |  |  |  |  |  |  | **0.53**** |
| **24 h after treatment** |  |  |  |  |  |  |  |  |  |  |  |
| **EVT alone** | | | | | | | | | | | |
| **Before treatment** | **MPO-DNA** | **0.57**** | **0.91**** | 0.15 | **0.46**** | 0.24 | -0.08 | 0.17 | 0.21 | **0.34*** | 0.29 |
| **1 h after** |  |  | **0.42*** | 0.08 | **0.43**** | -0.13 | -0.20 | -0.11 | 0.19 | 0.16 | -0.17 |
| **24 h after treatment** |  |  |  | '-0.09 | 0.33 | 0.13 | -0.32 | 0.07 | 0.31 | 0.27 | 0.17 |
| **Before treatment** | **Histone-DNA** |  |  |  | 0.12 | **0.69**** | **0.39**** | 0.45 | 0.14 | 0.10 | 0.32 |
| **1 h after** |  |  |  |  |  | 0.04 | **-0.41**** | -0.07 | 0.25 | 0.25 | -0.05 |
| **24 h after treatment** |  |  |  |  |  |  | 0.11 | **0.66**** | 0.15 | **0.42*** | 0.28 |
| **Before treatment** | **CitH3** |  |  |  |  |  |  | **0.36*** | -0.05 | -0.08 | **0.38*** |
| **24 h after treatment** |  |  |  |  |  |  |  |  | 0.16 | 0.36 | **0.38*** |
| **Before treatment** | **CRP** |  |  |  |  |  |  |  |  | **0.96**** | **0.64**** |
| **1 h after** |  |  |  |  |  |  |  |  |  |  | **0.68**** |
| **24 h after treatment** |  |  |  |  |  |  |  |  |  |  |  |

| **SUPPLEMENTARY Table S3. Association of NETs, inflammation, and coagulation biomarkers before heparin plus EVT or EVT alone treatment with baseline NIHSS scores.** | | | | |  |
| --- | --- | --- | --- | --- | --- |
|  |  |  |  |  |  |
| **Biomarkers** | **Baseline NIHSS scores** | | | |  |
|  | **Model 1** | | **Model 2** | |  |
|  | **β (95% CI)** | **p-Value** | **β (95% CI)** | **p-Value** |  |
| **NETs** | | | | |  |
| **MPO-DNA complexes (AU/uL)** | | | | |  |
| Before EVT+ LD-Hep treatment | 0.80 (-3.27, 4.86) | 0.70 | 0.96 (-3.29, 5.23) | 0.65 |  |
| Before EVT alone treatment | 1.79 (-2.56, 6.14) | 0.41 | 2.34 (-1.69, 6.36) | 0.25 |  |
| **Histone-DNA complexes (ng/mL)** | | | | |  |
| Before EVT+ LD-Hep treatment | -3.78 (-7.44, -0.13) | 0.04 | -1.86 (-5.61, 1.90) | 0.33 |  |
| Before EVT alone treatment | 2.12 (-2.03, 6.27) | 0.31 | 0.48 (-3.56, 4.52) | 0.81 |  |
| **CitH3 (ng/L)** | | | | |  |
| Before EVT+ LD-Hep treatment | **-0.47 (-0.94, 0.01)** | **0.06** | **-0.47 (-0.91, -0.03)** | **0.04** |  |
| Before EVT alone treatment | 3.90 (0.97, 6.84) | 0.01 | 1.40 (-1.98, 4.26) | 0.47 |  |
| **Inflammation** | | | | |  |
| **CRP (mg/L)** | | | | |  |
| Before EVT+ LD-Hep treatment | 0.02 (-0.09, 0.13) | 0.73 | 0.04 (-0.07, 0.14) | 0.48 |  |
| Before EVT alone treatment | 0.08 (-0.02, 0.17) | 0.10 | -0.01 (-0.10, 0.09) | 0.91 |  |
| **Coagulation** | | | | |  |
| **APTT (sec)** | | | | |  |
| Before EVT+ LD-Hep treatment | 0.02 (-0.01, 0.04) | 0.18 | 0.21 (-0.41, 0.82) | 0.50 |  |
| Before EVT alone treatment | -0.05 (-0.15, 0.05) | 0.29 | -0.63 (-3.13, 1.86) | 0.61 |  |
| Model1= unadjusted | | | | |  |
| Model2= adjusted for the diabetes mellitus, atrial fibrillation, age, sex, weight, baseline mRS, baseline ASPECTS, collateral status, time from onset to randomization. Linear regression was used to investigate the association between NETs, inflammation, and coagulation biomarkers and stroke severity before treatment. Abbreviation: Myeloperoxidase DNA complexes = MPO-DNA complexes, Citrullinated histone H3 = CitH3, C-reactive protein = CRP, Activated partial thromboplastin time = APTT, Endovascular thrombectomy alone = EVT alone treatment, Endovascular thrombectomy + heparin treatment = EVT+ LD-Hep treatment, National Institutes of Health Stroke Scale score = NIHSS score, Alberta Stroke Program Early CT Score = ASPECTS. | | | | |  |

| **SUPPLEMENTARY Table S4:** Association of NETs, inflammation, and coagulation biomarkers before heparin plus EVT or EVT alone treatment with baseline ASPECTS scores. | | | | |  |
| --- | --- | --- | --- | --- | --- |
|  |  |  |  |  |  |
| **Biomarkers** | **Baseline ASPECTS scores** | | | |  |
|  | **Model 1** | | **Model 2** | |  |
|  | **β (95% CI)** | **p-Value** | **β (95% CI)** | **p-Value** |  |
| **NETs** | | | | |  |
| **MPO-DNA complexes (AU/uL)** | | | | |  |
| Before EVT+ LD-Hep treatment | 0.01 (-0.09, 0.10) | 0.88 | -0.24 (-1.62, 1.14) | 0.73 |  |
| Before EVT alone treatment | 0.02 (-0.08, 0.04) | 0.54 | 0.03 (-0.99, 1.04) | 0.56 |  |
| **Histone-DNA complexes (ng/mL)** | | | | |  |
| Before EVT+ LD-Hep treatment | 0.01 (-0.01, 0.10) | 0.85 | 0.04 (-1.20, 1.28) | 0.95 |  |
| Before EVT alone treatment | 0.01 (-0.08, 0.10) | 0.84 | -0.11 (-1.05, 0.83) | 0.82 |  |
| **CitH3 (ng/L)** |  | | | |  |
| Before EVT+ LD-Hep treatment | 0.00 (-0.01, 0.02) | 0.60 | 0.05 (-0.10, 0.20) | 0.52 |  |
| Before EVT alone treatment | 0.00 (-0.01, 0.02) | 0.60 | **-0.72 (-1.44, -0.01)** | **0.05** |  |
| **Inflammation** | | | | |  |
| **CRP (mg/L)** | | | | |  |
| Before EVT+ LD-Hep treatment | -0.00 (-0.00, 0.00) | 0.57 | -0.01 (-0.05, 0.02) | 0.40 |  |
| Before EVT alone treatment | -0.00 (-0.00, 0.00) | 0.57 | -0.01 (-0.03, 0.02) | 0.49 |  |
| **Coagulation** | | | | |  |
| **APTT (sec)** | | | | |  |
| Before EVT+ LD-Hep treatment | -0.01(-0.02, 0.01) | 0.39 | **-0.21 (-0.39, -0.03)** | **0.03** |  |
| Before EVT alone treatment | -0.01 (-0.02, 0.01) | 0.39 | -0.28 (-0.89, 0.33) | 0.36 |  |
| Model1= unadjusted | | | | |  |
| Model2= adjusted for the diabetes mellitus, atrial fibrillation, age, sex, weight, baseline mRS, baseline NIHSS score, collateral status, time from onset to randomization. Linear regression was used to investigate the association between NETs, inflammation, and coagulation biomarkers and baseline ASPECTS scores. Abbreviation: Myeloperoxidase DNA complexes = MPO-DNA complexes, Citrullinated histone H3 = CitH3, C-reactive protein = CRP, Activated partial thromboplastin time = APTT, Endovascular thrombectomy alone = EVT alone treatment, Endovascular thrombectomy + heparin treatment = EVT+ LD-Hep treatment, National Institutes of Health Stroke Scale score = NIHSS score, Alberta Stroke Program Early CT Score = ASPECTS. | | | | |  |

| **SUPPLEMENTARY Table S5:** Association of NETs, inflammation, and coagulation biomarkers before, 1 h, and 24 h after heparin plus EVT or EVT alone treatment with final infarct size at 7 days after treatment in AIS patients. | | | | |
| --- | --- | --- | --- | --- |
|  |  |  |  |  |
| **Biomarkers** | **Final infarct size** | | | |
|  | **Model 1** | | **Model 2** | |
|  | **β (95% CI)** | **p-Value** | **β (95% CI)** | **p-Value** |
| **NETs** | | | | |
| **MPO-DNA complexes (AU/uL)** | | | | |
| Before EVT+ LD-Hep treatment | 0.09 (-0.39, 0.58) | 0.71 | 0.12 (-0.43, 0.66) | 0.66 |
| 1 h after EVT+ LD-Hep | **-0.41 (-0.79, -0.03)** | **0.03** | -0.22 (-0.59, 0.14) | 0.23 |
| 24 h after EVT+ LD-Hep | -0.37 (-0.99, 0.26) | 0.25 | -0.05 (-0.74, 0.65) | 0.88 |
| Before EVT alone treatment | 0.05 (-0.60, 0.49) | 0.85 | -0.24 (-0.75, 0.28) | 0.36 |
| 1 h after EVT alone | -0.21 (-0.64, 0.22) | 0.33 | -0.45 (-0.92, 0.02) | 0.06 |
| 24 h after EVT alone | 0.00 (-064, 0.64) | 1.00 | -0.16 (-0.80, 0.48) | 0.61 |
| **Histone-DNA complexes (ng/mL)** | | | | |
| Before EVT+ LD-Hep treatment | 0.11 (-0.36, 0.58) | 0.64 | 0.18 (-0.33, 0.70) | 0.48 |
| 1 h after EVT+ LD-Hep | 0.27 (-0.19, 0.73) | 0.25 | 0.46 (0.03, 0.89) | 0.04 |
| 24 h after EVT+ LD-Hep | -0.61 (-1.22, 0.01) | 0.05 | -0.27 (-0.33, 1.29) | 0.23 |
| Before EVT alone treatment | 0.03 (-0.51, 0.56) | 0.92 | -0.22 (-0.69, 0.26) | 0.36 |
| 1 h after EVT alone | 0.47 (-0.51, 0.61) | 0.17 | -0.07 (-0.67, 0.53) | 0.82 |
| 24 h after EVT alone | -0.37 (-1.18, 0.43) | 0.35 | 0.17 (-0.74, 1.08) | 0.69 |
| **CitH3 (ng/L)** | | | | |
| Before EVT+ LD-Hep treatment | -0.03 (-0.09, 0.03) | 0.39 | -0.01 (-0.07, 0.05) | 0.81 |
| 24 h after EVT+ LD-Hep | - 0. 01 (-0.07, 0.05) | 0.74 | -0.01 (-0.07, 0.05) | 0.73 |
| Before EVT alone treatment | 0.27 (-0.13, 0.68) | 0.18 | 0.01 (-0.39, 0.42) | 0.95 |
| 24 h after EVT alone | -0.04 (-0.51, 0.42) | 0.86 | 0.39 (-0.40, 1.18) | 0.31 |
| **Inflammation** | | | | |
| **CRP (mg/L)** | | | | |
| Before EVT+ LD-Hep treatment | 0.01 (-0.01, 0.02) | 0.35 | 0.00 (-0.01, 0.02) | 0.58 |
| 1 h after EVT+ LD-Hep | -0.01 (-0.02, 0.01) | 0.40 | -0.00 (-0.02, 0.01) | 0.74 |
| 24 h after EVT+ LD-Hep | 0.01 (-0.00, 0.02) | 0.07 | 0.01 (-0.01, 0.02) | 0.34 |
| Before EVT alone treatment | 0.00 (-0.01, 0.02) | 0.29 | -0.01 (-0.02, 0.01) | 0.47 |
| 1 h after EVT alone | -0.00 (-0.02, 0.02) | 0.93 | -0.01 (-0.03, 0.02) | 0.48 |
| 24 h after EVT alone | 0.01 (-0.00, 0.02) | 0.16 | 0.01 (-0.01, 0.02) | 0.54 |
| **Coagulation** | | | | |
| **APTT (sec)** | | | | |
| Before EVT+ LD-Hep treatment | -0.02 (-0.09, 0.05) | 0.61 | -0.04 (-0.11, 0.04) | 0.30 |
| 1 h after EVT+ LD-Hep | 0.01 (-0.05, 0.07) | 0.64 | -0.01 (-0.07, 0.05) | 0.74 |
| Before EVT alone treatment | -0.13 (-0.42, 0.17) | 0.39 | -0.01 (-0.32, 0.29) | 0.94 |
| 1 h after EVT alone | -0.21 (-0.54, 0.12) | 0.21 | -0.06 (-0.41, 0.28) | 0.71 |
| **Anti_Xa (-)** | | | | |
| 1 h after EVT+ LD-Hep | -0.22 (-0.68, 0.24) | 0.35 | -0.33 (-0.24, 0.14) | 0.16 |
| Model 1= unadjusted | | | | |
| Model 2= Adjusted for the diabetes mellitus, atrial fibrillation, age, sex, weight, baseline mRS, collateral status, time from onset to randomization, baseline ASPECTS, baseline NIHSS score. Linear regression was used to investigate the association between NETs, inflammation, and coagulation biomarkers and final infarct size. Abbreviation: Myeloperoxidase DNA complexes = MPO-DNA complexes, Citrullinated histone H3 = CitH3, C-reactive protein = CRP, Activated partial thromboplastin time = APTT, Endovascular thrombectomy alone = EVT alone treatment, Endovascular thrombectomy + heparin treatment = EVT+ LD-Hep treatment, National Institutes of Health Stroke Scale score = NIHSS score, National Institutes of Health Stroke Scale score = NIHSS score, Alberta Stroke Program Early CT Score = ASPECTS. | | | | |

| **SUPPLEMENTARY Table S6: linear association between NETs markers and clinical or radiological outcomes, using** **Pearson's correlation coefficient.** | | | | | | | |
| --- | --- | --- | --- | --- | --- | --- | --- |
| **Blood collection time point** | **Biomarkers** | **Outcome after treatment** | | | **Baseline outcome** | | |
|  |  | **at 90-day** | **at 24 h** | **at 5-7 days** | **NIHSS** | **mRS** | **ASPECTS** |
|  |  | **mRS scores (0 - 6)** | **NIHSS** | **Final infarct size** |  |  |  |
| **Heparin+EVT** | | | | | | | |
| **Before treatment** | **MPO-DNA** | 0.08 | 0.03 | 0.05 | 0.04 | -0.06 | 0.03 |
| **1 h after** |  | 0.08 | -0.12 | **-0.27*** | 0.01 | -0.02 | 0.11 |
| **24 h after treatment** |  | -0.27 | -0.13 | -0.19 | -0.03 | -0.13 | -0.03 |
| **Before treatment** | **Histone-DNA** | 0.13 | 0.01 | 0.08 | **-0.28** | 0.15 | -0.12 |
| **1 h after** |  | 0.21 | 0.16 | 0.21 | 0.03 | 0.14 | -0.10 |
| **24 h after treatment** |  | **-0.36** | **-0.42** | **-0.34*** | -0.11 | 0.01 | 0.04 |
| **Before treatment** | **CitH3** | 0.04 | 0.09 | 0.14 | 0.08 | 0.12 | -0.05 |
| **24 h after treatment** |  | -0.25 | -0.08 | -0.02 | 0.09 | -0.17 | -0.05 |
| **Before treatment** | **CRP** | 0.10 | 0.12 | 0.02 | 0.19 | 0.11 | -0.15 |
| **1 h after** |  | 0.02 | 0.01 | -0.18 | 0.01 | 0.12 | -0.15 |
| **24 h after treatment** |  | 0.02 | 0.02 | **0.35*** | 0.08 | 0.13 | **-0.31*** |
| **EVT alone** | | | | | | | |
| **Before treatment** | **MPO-DNA** | 0.03 | -0.01 | -0.05 | 0.10 | **0.25*** | 0.02 |
| **1 h after** |  | 0.004 | -0.08 | -0.16 | 0.02 | 0.12 | 0.05 |
| **24 h after treatment** |  | 0.10 | 0.08 | 0.03 | 0.21 | 0.13 | -0.08 |
| **Before treatment** | **Histone-DNA** | 0.11 | 0.12 | 0.03 | 0.15 | 0.13 | -0.02 |
| **1 h after** |  | 0.01 | -0.06 | 0.09 | -0.03 | 0.23 | 0.01 |
| **24 h after treatment** |  | 0.23 | 0.20 | -0.17 | 0.09 | 0.28 | 0.09 |
| **Before treatment** | **CitH3** | 0.15 | **0.28*** | 0.15 | **0.36**** | 0.10 | -0.17 |
| **24 h after treatment** |  | 0.02 | -0.08 | -0.08 | 0.02 | 0.30 | 0.19 |
| **Before treatment** | **CRP** | 0.21 | 0.10 | 0.12 | 0.16 | **0.52**** | 0.08 |
| **1 h after** |  | 0.18 | 0.13 | 0.10 | 0.09 | 0.54** | -0.03 |
| **24 h after treatment** |  | **0.32*** | 0.18 | 0.23 | 0.19 | **0.44**** | -0.18 |

| **SUPPLEMENTARY Table S7:** Association of NETs, inflammation, and coagulation biomarkers before, 1 h, and 24 h after heparin plus EVT or EVT alone treatment with NIHSS scores at 24h after treatment in AIS patients. | | | | |  |
| --- | --- | --- | --- | --- | --- |
|  |  |  |  |  |  |
| **Biomarkers** | **NIHSS scores** | | | |  |
|  | **Model 1** | | **Model 2** | |  |
|  | **β (95% CI)** | **p-Value** | **β (95% CI)** | **p-Value** |  |
| **NETs** | | | | |  |
| **MPO-DNA complexes (AU/uL)** | | | | |  |
| Before EVT+ LD-Hep treatment | -0.09 (-1.13, 0.95) | 0.86 | 0.24 (-0.84, 1.13) | 0.66 |  |
| 1 h after EVT+ LD-Hep | -0.34 (-1.23, 0.55) | 0.45 | 0.25 (-0.67, 1.17) | 0.59 |  |
| 24 h after EVT+ LD-Hep | -0.41 (-1.69, 0.87) | 0.52 | 0.21 (-1.48, 1.91) | 0.80 |  |
| Before EVT alone treatment | -0.64 (-1.69, 0.40) | 0.22 | -0.68 (-1.67, 0.32) | 0.18 |  |
| 1 h after EVT alone | -0.21 (-1.01, 0.59) | 0.60 | -0.56 (-1.39, 0.27) | 0.18 |  |
| 24 h after EVT alone | 0.31 (-0.83, 1.45) | 0.59 | -0.34 (-1.45, 0.76) | 0.53 |  |
| **Histone-DNA complexes (ng/mL)** | | | | |  |
| Before EVT+ LD-Hep treatment | 0.05 (-0.934, 1.05) | 0.92 | 0.01 (-0.97, 0.99) | 0.99 |  |
| 1 h after EVT+ LD-Hep | 0.48 (-0.59, 1.55) | 0.38 | 0.84 (-0.31, 2.00) | 0.13 |  |
| 24 h after EVT+ LD-Hep | -1.54 (-2.66, -04) | 0.01 | -1.34 (-2,76, 0,08) | 0.06 |  |
| Before EVT alone treatment | 0.26 (-0.67, 1.19) | 0.60 | -0.13 (-1.01, 0.76) | 0.78 |  |
| 1 h after EVT alone | -0.48 (-1.33, 0.64) | 0.48 | -0.38 (-1.32, 0.58) | 0.43 |  |
| 24 h after EVT alone | 0.70 (-0,48, 1.87) | 0.24 | 0.75 (-0.39, 1.89) | 0.19 |  |
| **CitH3 (ng/L)** | | | | |  |
| Before EVT+ LD-Hep treatment | -0.10 (-0.23, 0.03) | 0.14 | -0.05 (-0.17, 0.07) | 0.41 |  |
| 24 h after EVT+ LD-Hep | -0.05 (-0.18, 0.07) | 0.40 | -0.02 (-0.16, 0.13) | 0.80 |  |
| Before EVT alone treatment | 0.71 (0.04, 1.39) | 0.04 | 0.17 (-0.57, 0.91), 58 | 0.65 |  |
| 24 h after EVT alone | 0.71 (0.04, 1.39) | 0.04 | -0.42 (-1.61, 0.77), 39 | 0.48 |  |
| **Inflammation** | | | | |  |
| **CRP (mg/L)** | | | | |  |
| Before EVT+ LD-Hep treatment | 0.02 (-0.01, 0.05) | 0.19 | 0.02 (-0.01, 0.05) | 0.15 |  |
| 1 h after EVT+ LD-Hep | -0.01 (-0.05, 0.03) | 0.57 | -0.01 (-0.05, 0.03) | 0.53 |  |
| 24 h after EVT+ LD-Hep | 0.00 (-0.02, 0.03) | 0.88 | -0.02 (-0.04, 0.02) | 0.39 |  |
| Before EVT alone treatment | -0.00 (-0.04, 0.03) | 0.88 | -0.03 (-0.07, 0.00) | 0.08 |  |
| 1 h after EVT alone | -0.00 (-0.03, 0.03) | 0.94 | -0.02 (-0.06, 0.02) | 0.29 |  |
| 24 h after EVT alone | **0.02 (0.00, 0.05)** | **0.03** | 0.01 (-0.02, 0.03) | 0.66 |  |
| **Coagulation** | | | | |  |
| **APTT (sec)** | | | | |  |
| Before EVT+ LD-Hep treatment | -0.04 (-0.18, 0.11) | 0.62 | -0.06 (-0.22, 0.10) | 0.47 |  |
| 1 h after EVT+ LD-Hep | -0.08 (-0.22, 0.06) | 0.27 | -0.08 (-0.24, 0.08) | 0.30 |  |
| Before EVT alone treatment | -0.35 (-0.92, 0.21) | 0.22 | -0.15 (-0.76, 0.46) | 0.62 |  |
| 1 h after EVT alone | -0.38 (-1.05, 0.28) | 0.26 | 0.10 (-0.55, 0.75) | 0.76 |  |
| **Anti_Xa, n** |  |  |  |  |  |
| 1 h after EVT+ LD-Hep | -0.44 (-1.47, 0.59) | 0.40 | -0.59 (-1.77, 0.58) | 0.32 |  |
| Model1= unadjusted | | | | |  |
| Model 2= Adjusted for the diabetes mellitus, atrial fibrillation, age, sex, weight, baseline mRS, collateral status, time from onset to randomization, baseline ASPECTS, baseline NIHSS score. Linear regression was used to investigate the association between NETs, inflammation, and coagulation biomarkers and NIHSS scores at 24 h post-reperfusion. Abbreviation: Myeloperoxidase DNA complexes = MPO-DNA complexes, Citrullinated histone H3 = CitH3, C-reactive protein = CRP, Activated partial thromboplastin time = APTT, Endovascular thrombectomy alone = EVT alone treatment, Endovascular thrombectomy + heparin treatment = EVT+ LD-Hep treatment, National Institutes of Health Stroke Scale score = NIHSS score, Alberta Stroke Program Early CT Score = ASPECTS. | | | | |  |

| **SUPPLEMENTARY Table S8:** Association of NETs, inflammation, and coagulation biomarkers before, 1 h, and 24 h after heparin plus EVT or EVT alone treatment with unfavorable long-term fuctional outcome (3-6 mRS) at 90 days after treatment in AIS patients. | | | | |
| --- | --- | --- | --- | --- |
|  |  |  |  |  |
| **Biomarkers** | **mRS score (unfavorable ≥3)** | | | |
|  | **Model 1** | | **Model 2** | |
|  | **ODDs (95% CI)** | **p-Value** | **ODDs (95% CI)** | **p-Value** |
| **NETs** | | | | |
| **MPO-DNA complexes (AU/uL)** | | | | |
| Before EVT+ LD-Hep treatment | 1.55 (0.44, 5.41) | 0.50 | 5.11 (0.63, 41.53) | 0.13 |
| 1 h after EVT+ LD-Hep | 0.89 (0.35, 2.29) | 0.81 | 0.92 (0.25, 3.45) | 0.90 |
| 24 h after EVT+ LD-Hep | 1.09 (0.23, 5.24) | 0.92 | 1.66 (0.16, 17.03) | 0.67 |
| Before EVT alone treatment | 0.54 (0.16, 1.81) | 0.32 | 0.43 (0.08, 2.35) | 0.33 |
| 1 h after EVT alone | 1.04 (0.36, 2.95) | 0.94 | 0.82 (0.14, 4.66) | 0.82 |
| 24 h after EVT alone | 0.80 (0.20, 3.25) | 0.76 | 1.07 (0.98, 1.16) | 0.12 |
| **Histone-DNA complexes (ng/mL)** | | | | |
| Before EVT+ LD-Hep treatment | 2.28 (0.69, 7.54) | 0.18 | 2.76 (0.51, 14.79) | 0.24 |
| 1 h after EVT+ LD-Hep | 3.04 (0.93, 9.95) | 0.07 | 4.79 (0.81, 28.28) | 0.08 |
| 24 h after EVT+ LD-Hep | 0.13 (0.03, 0.64) | 0.02 | 0.01 (0.00, 1.39) | 0.08 |
| Before EVT alone treatment | 0.77 (0.23, 2.60) | 0.12 | 0.35 (0.05, 2.37) | 0.28 |
| 1 h after EVT alone | 0.66 (0.18, 2.44) | 0.54 | 0.25 (0.03, 2.25) | 0.22 |
| 24 h after EVT alone | 2.17 (0.40, 11.74) | 0.37 | 1.18 (0.07, 20.25) | 0.23 |
| **CitH3 (ng/L)** | | | | |
| Before EVT+ LD-Hep treatment | 0.54 (0.52, 1.40) | 0.54 | 0.84 (0.25, 2.76) | 0.77 |
| 24 h after EVT+ LD-Hep | 0.69 (0.26, 1.86) | 0.47 | 0.95 (0.59, 1.54) | 0.84 |
| Before EVT alone treatment | 1.13 (0.49, 2.63) | 0.77 | 0.49 (0.12, 1.99) | 0.32 |
| 24 h after EVT alone | 1.58 (0.51, 4.89) | 0.43 | 0.09 (0.003, 2.68) | 0.67 |
| **Inflammation** | | | | |
| **CRP (mg/L)** | | | | |
| Before EVT+ LD-Hep treatment | 1.03 (0.97, 1.09) | 0.34 | 1.01 (0.97, 1.06) | 0.52 |
| 1 h after EVT+ LD-Hep | 0.98 (0.94, 1.03) | 0.49 | 0.96 (0.89, 1.04) | 0.28 |
| 24 h after EVT+ LD-Hep | 0.99 (0.95, 1.02) | 0.45 | 0.88 (0.76, 1.03) | 0.12 |
| Before EVT alone treatment | 1.04 (1.00, 1.09) | 0.08 | 1.00 (0.95, 1.06) | 0.88 |
| 1 h after EVT alone | 1.01 (), 0.97, 1.05) | 0.59 | 0.95 (0.87, 1.04) | 0.27 |
| 24 h after EVT alone | 1.06 (1.00, 1.12) | 0.05 | 0.81 (0.57, 1.16) | 0.25 |
| **Coagulation** | | | | |
| **APTT (sec)** | | | | |
| Before EVT+ LD-Hep treatment | 0.97 (0.99, 1.01) | 0.97 | 1.02 (0.79, 1.31) | 0.89 |
| 1 h after EVT+ LD-Hep | 1.00 (1.00, 1.01) | 0.49 | 1.08 (0.87, 1.34) | 0.48 |
| Before EVT alone treatment | 0.86 (0.42, 1.75) | 0.68 | 0.84 (0.23, 3.04) | 0.80 |
| 1 h after EVT alone | 0.49 (0.11, 2.18) | 0.35 | 0.95 (0.19, 4.83) | 0.95 |
| **Anti_Xa (-)** | | | | |
| 1 h after EVT+ LD-Hep | 1.12 (0.38, 3.26) | 0.84 | 0.85 (0.18, 4.09) | 0.84 |
| Model 1 = unadjusted | | | | |
| Model 2 = Adjusted for the diabetes mellitus, atrial fibrillation, age, sex, weight, baseline mRS, collateral status, time from onset to randomization, baseline ASPECTS, baseline NIHSS score. Logistic regression was used to investigate the association between NETs, inflammation, and coagulation biomarkers and final infarct size. Abbreviation: Myeloperoxidase DNA complexes = MPO-DNA complexes, Citrullinated histone H3 = CitH3, C-reactive protein = CRP, Activated partial thromboplastin time = APTT, Endovascular thrombectomy alone = EVT alone treatment, Endovascular thrombectomy + heparin treatment = EVT+ LD-Hep treatment, National Institutes of Health Stroke Scale score = NIHSS score, Alberta Stroke Program Early CT Score = ASPECTS | | | | |


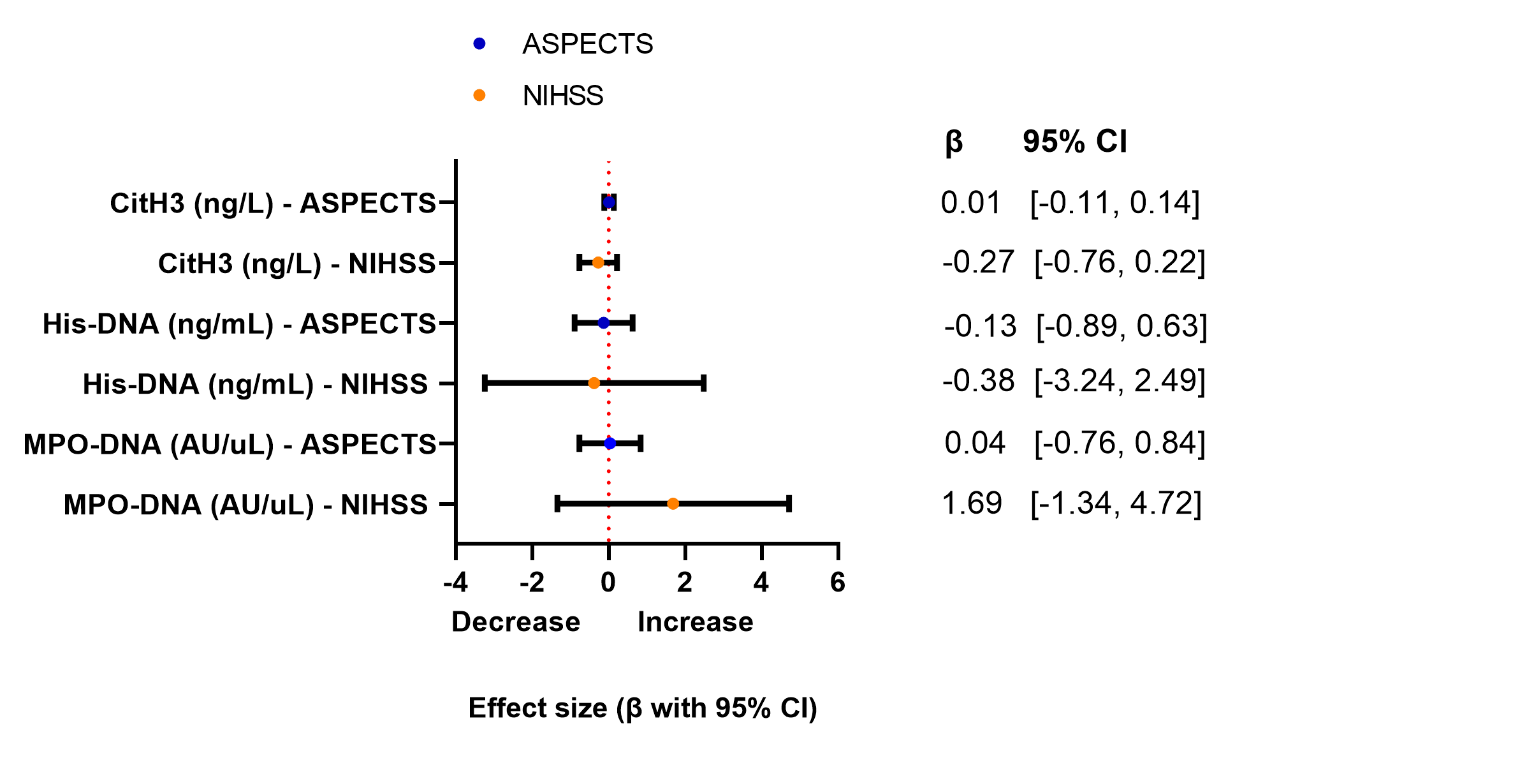


Figure S1: Association of NETs markers levels before heparin plus EVT or EVT alone treatment with stroke severity (NIHSS scores) and brain damage (ASPECTS) on admission. The Forest plot shows the β effect sizes with 95% CIs from the linear regression analysis for the associations between markers and baseline NIHSS scores as well as baseline ASPECTS. β and 95% CI are adjusted for diabetes mellitus, atrial fibrillation, age, sex, weight, collateral status, and time from onset to randomization. Abbreviation: Myeloperoxidase DNA complexes = MPO-DNA complexes, Citrullinated histone H3 = CitH3, National Institutes of Health Stroke Scale score = NIHSS score, Alberta Stroke Program Early CT Score = ASPECTS, Confidence interval = CI, Beta = β.

**Acknowledgement:**

**MR CLEAN-MED Investigators & Collaborators**

**Principal investigators**

Diederik Dippel (MD, PhD),^1^ Aad van der Lugt (MD, PhD)^1^

**Study coordinators**

Wouter van der Steen (MD),^1^ Rob van de Graaf (MD, PhD),^1^ Bob Roozenbeek (MD, PhD)^1^

**Local principal investigators**

Bob Roozenbeek (MD, PhD),^1^ Adriaan van Es (MD, PhD),^1,2^ Pieter Jan van Doormaal (MD),^1^ Jonathan M. Coutinho (MD, PhD),^3^ Bart Emmer (MD, PhD),^3^ Inger de Ridder (MD, PhD),^4^ Wim van Zwam (MD, PhD),^4^ Bart van der Worp (MD, PhD),^5^ Irene van der Schaaf (MD, PhD),^5^ Rob Gons (MD, PhD),^6^ Lonneke Yo (MD),^6^ Jelis Boiten (MD, PhD),^7^ Ido van den Wijngaard (MD, PhD),^2,7^ Jeanette Hofmeijer (MD, PhD),^8^ Jasper Martens (MD),^8^ Wouter Schonewille (MD, PhD),^9^ Jan Albert Vos, (MD, PhD),^9^ Anil Man Tuladhar (MD, PhD),^10^ Sjoerd Jenniskens (MD),^10^ Karlijn de Laat (MD, PhD),^11^ Lukas van Dijk (MD, PhD),^11^ Heleen den Hertog (MD, PhD),^12^ Boudewijn van Hasselt (MD),^12^ Michel Remmers (MD),^13^ Douwe Vos,^13^ Anouk Rozeman (MD, PhD),^14^ Otto Elgersma (MD, PhD),^14^ Maarten Uyttenboogaart (MD, PhD),^15^ Reinoud Bokkers (MD, PhD),^15^ Julia van Tuijl (MD, PhD),^16^ Issam Boukrab (MD),^16^ Benjamin Gory (MD, PhD),^17^ Arturo Consoli (MD),^18^ Mikaël Mazighi (MD, PhD),^19^ Frederic Clarencon (MD, PhD),^20^ Gaultier Marnat (MD),^21^

**Local trial collaborators:**

**Executive and writing committee**

Diederik Dippel (MD, PhD),^1^ Aad van der Lugt (MD, PhD),^1^ Rob van de Graaf (MD, PhD),^1^ Wouter van der Steen (MD),^1^ Bob Roozenbeek (MD, PhD),^1^ Adriaan van Es (MD, PhD),^1^ Yvo Roos (MD, PhD),^3^ Charles Majoie (MD, PhD),^3^ Robert van Oostenbrugge (MD, PhD),^4^ Wim van Zwam (MD, PhD),^4^ Julie Staals (MD, PhD),^4^ Sjoerd Jenniskens (MD),^10^ Lukas van Dijk (MD, PhD),^11^ Heleen den Hertog (MD, PhD),^12^

**Data Safety Monitoring Board**

Peter Rothwell (MD, PhD) *– Chair,*^22^ Andrew Molyneux (MD, PhD),^22^ Joanna Moschandreas (MD, PhD)^22^

**Independent trial statistician**

Daan Nieboer (MSc)^1^

**CONTRAST clinical trial collaborators:**

**Research leaders**

Diederik Dippel (MD, PhD),^1^ Charles Majoie (MD, PhD)^3^

**Consortium coordinator:**

Rick van Nuland, (PhD)^24^

**Imaging assessment committee**

Charles Majoie (MD, PhD) *– Chair*,^3^ Aad van der Lugt (MD, PhD) *– Chair*,^1^ Adriaan van Es, (MD, PhD),^1,2^ Pieter-Jan van Doormaal (MD),^1^ René van den Berg, (MD, PhD),^3^ Ludo Beenen (MD, PhD),^3^ Bart Emmer (MD, PhD),^3^ Stefan Roosendaal (MD, PhD),^3^ Wim van Zwam (MD, PhD),^4^ Alida Annechien Postma (MD, PhD),^25^ Lonneke Yo (MD, PhD),^6^ Menno Krietemeijer (MD),^6^ Geert Lycklama (MD, PhD),^7^ Jasper Martens (MD),^8^ Sebastiaan Hammer (MD, PhD),^10^ Anton Meijer (MD, PhD),^10^ Reinoud Bokkers (MD, PhD),^15^ Anouk van der Hoorn (MD, PhD),^15^ Ido van den Wijngaard (MD, PhD),^2,7^ Albert Yoo (MD, PhD),^26^ Dick Gerrits (MD)^27^

**Adverse event committee**

Robert van Oostenbrugge (MD, PhD) *– Chair*,^4^ Bart Emmer (MD, PhD),^3^ Jonathan M. Coutinho (MD, PhD),^3^ Martine Truijman (MD, PhD),^4^ Julie Staals (MD, PHD),^4^ Bart van der Worp (MD, PhD),^5^ J. Boogaarts (MD, PhD),^10^ Ben Jansen (MD, PhD),^16^ Sanne Zinkstok (MD, PhD)^28^

**Outcome assessment committee**

Yvo Roos (MD, PhD) – Chair,^1^ Sanne Manschot (MD, PhD),^7^ Diederik Dippel (MD, PhD),^2^ Henk Kerkhof (MD, PhD),^15^ Ido van den Wijngaard (MD, PhD),^7^ Jonathan Coutinho (MD, PhD),^1^ Peter Koudstaal (MD, PhD),^1^ Koos Keizer (MD, PhD)^6^, Jelis Boiten (MD, PhD)^7^

**Data management group**

Hester Lingsma (PhD),^1^ Diederik Dippel (MD, PhD),^1^ Vicky Chalos (MD),^1^ Olvert Berkhemer (MD, PhD)^1,3^

**Imaging data management**

Aad van der Lugt (MD, PhD),^1^ Charles Majoie (MD, PhD),^3^ Adriaan Versteeg,^1^ Lennard Wolff (MD),^1^ Matthijs van der Sluijs (MD),^1^ Henk van Voorst (MD),^3^ Manon Tolhuisen (MSc),^3^

**Biomaterials and translational group**

Hugo ten Cate (MD, PhD),^4^ Moniek de Maat (PhD),^1^ Samantha Donse-Donkel (MD),^1^ Aarazo Barakzie (MSc),^1^ Heleen van Beusekom (PhD),^1^ Aladdin Taha (MD),^1^

**Local collaborators**

Vicky Chalos (MD, PhD),^1^ Rob van de Graaf (MD, PhD),^1^ Wouter van der Steen (MD),^1^ Aladdin Taha (MD),^1^ Samantha Donse-Donkel (MD),^1^ Lennard Wolff (MD),^1^ Kilian Treurniet (MD),^3^ Sophie van den Berg (MD),^3^ Natalie LeCouffe (MD),^3^ Manon Kappelhof (MD),^3^ Rik Reinink (MD),^3^ Manon Tolhuisen (MD),^3^ Leon Rinkel (MD),^3^ Josje Brouwer (MD),^3^ Agnetha Bruggeman (MD),^3^ Henk van Voorst (MD),^3^ Robert-Jan Goldhoorn (MD),^4^ Wouter Hinsenveld (MD),^4^ Anne Pirson (MD),^4^ Susan Olthuis (MD),^4^ Simone Uniken Venema (MD),^5^ Sjan Teeselink (MD),^10^ Lotte Sondag (MD),^10^ Sabine Collette (MD)^15^

**Research nurses**

Martin Sterrenberg,^1^ Naziha El Ghannouti,^1^ Laurine van der Steen,^3^ Sabrina Verheesen,^4^ Jeannique Vranken,^4^ Ayla van Ahee,^5^ Hester Bongenaar,^6^ Maylee Smallegange,^6^ Lida Tilet,^6^ Joke de Meris,^7^ Michelle Simons,^8^ Wilma Pellikaan,^9^ Wilma van Wijngaarden,^9^ Kitty Blauwendraat,^9^ Yvonne Drabbe,^11^ Michelle Sandiman-Lefeber,^11^ Anke Katthöfer,^11^ Eva Ponjee,^12^ Rieke Eilander,^12^ Anja van Loon,^13^ Karin Kraus,^13^ Suze Kooij,^14^ Annemarie Slotboom,^14^ Marieke de Jong,^15^ Friedus van der Minne,^15^ Esther Santegoets^16^

**Study monitors**

Leontien Heiligers^1^, Yvonne Martens,^1^ Naziha El Ghannouti^1^

##### **Affiliations**

1. Erasmus MC University Medical Center, Rotterdam, the Netherlands
2. Leiden University Medical Center, Leiden, the Netherlands
3. Amsterdam University Medical Centers, location AMC, Amsterdam, the Netherlands
4. Cardiovascular Research Institute Maastricht (CARIM), Maastricht University Medical Centre, Maastricht, The Netherlands
5. University Medical Center Utrecht, Brain Center Rudolf Magnus, Utrecht, the Netherlands
6. Catharina Hospital, Eindhoven, the Netherlands
7. Haaglanden Medical Centre, the Hague, the Netherlands
8. Rijnstate Hospital, Arnhem, the Netherlands
9. St. Antonius Hospital, Nieuwegein, the Netherlands
10. Radboud University Medical Center, Nijmegen, the Netherlands
11. HagaZiekenhuis, the Hague, the Netherlands
12. Isala, Zwolle, the Netherlands
13. Amphia Hospital, Breda, the Netherlands
14. Albert Schweitzer Hospital, Dordrecht, the Netherlands
15. University Medical Center Groningen, Groningen, the Netherlands
16. Elisabeth-TweeSteden Hospital, Tilburg, the Netherlands;
17. University Hospital of Nancy, Nancy, France
18. Foch Hospital, Suresnes, France
19. Fondation Rothschild Hospital, Paris, France
20. University Hospital of Bordeaux, Bordeaux, France
21. Pitié-Salpêtrière University hospital, Paris, France
22. John Radcliffe Hospital, Oxford, United Kingdom
23. University of Washington, Seattle, Washington, United States
24. Lygature, Utrecht, the Netherlands;
25. School for Mental Health and Sciences (Mhens), Maastricht University Medical Center, Maastricht, The Netherlands
26. Texas Stroke Institute, Dallas-Fort Worth, Texas, United States of America
27. Medisch Spectrum Twente, Enschede, The Netherlands
28. TerGooi, Hilversum, The Netherlands
